# Self-Directed Neurofeedback Treatment for Subjective Tinnitus Patients Evaluated by Multimodal Functional Imaging

**DOI:** 10.1155/2022/5114721

**Published:** 2022-10-18

**Authors:** Xiaoyan Ma, Fangyuan Wang, Chi Zhang, Weidong Shen, Shiming Yang

**Affiliations:** ^1^The First Affiliated Hospital of Xi'an Jiaotong University, Shanxi, China; ^2^Senior Department of Otolaryngology-Head & Neck Surgery, The Sixth Medical Center of PLA General Hospital, Beijing, China; ^3^National Clinical Research Center for Otolaryngologic Diseases, Beijing, China; ^4^State Key Lab of Hearing Science, Ministry of Education, China; ^5^Beijing Key Lab of Hearing Impairment Prevention and Treatment, Beijing, China

## Abstract

Neurofeedback (NFB) is a relatively novel approach to the treatment of tinnitus, and prior studies have demonstrated that the increases in alpha activity rather than reduced delta power seem to drive these NFB-related improvements in tinnitus symptoms. The present study was therefore designed to explore whether the implementation of an alpha training protocol with a portable neurofeedback apparatus would achieve improvements in tinnitus patient symptoms. In this study, 38 tinnitus patients underwent NFB training while 18 were enrolled in a control group. The study was single-blinded such that only participants were not aware of their group assignments. Those in the NFB group underwent 15 NFB training sessions over 5 weeks, in addition to pre- and posttraining tests including the Tinnitus Handicap Inventory (THI), Tinnitus Handicap Questionnaire (THQ), visual analog scales (VAS), electroencephalography (EEG), and functional magnetic resonance imaging (fMRI). Our result find that when the THI, THQ, and VAS scores of patients in the two groups were assessed after a 5-week training period, these scores were unchanged in control patients whereas they had significantly improved in the NFB group patients. EEG analyses revealed that the alpha band was increased in the occipital lobe following NFB treatment, while fMRI indicated an increase in regional homogeneity (ReHo) in the right frontal lobe of patients in the NFB group after treatment that was negatively correlated with THI and VAS scores. The results of this analysis indicate that alpha NFB training can be effectively used to reduce tinnitus-related distress and sound perception in patients.

## 1. Introduction

Tinnitus is a condition wherein affected individuals perceive sounds that do not correspond to any external acoustic source [1, 2], with symptoms of this condition generally manifesting as unilateral, bilateral, or disseminated perceptions of a particular pitch or noise [3]. Tinnitus can also result in complications such as difficulty sleeping, anxiety, depression, and impaired social interaction [4, 5]. Reliable approaches to alleviating the underlying symptoms of tinnitus have not been developed to date, and many affected individuals feel they do not require assistance. This can lead to chronic increases in stress levels for tinnitus sufferers, ultimately compounding the effects of this condition and markedly reducing patient quality of life [6].

The neurophysiological basis for tinnitus is not well understood, although it has been considered correlate with cochlear damage [7]. Damage to cochlear hair cells can deprive auditory neurons of appropriate input [8], resulting in cortical map reorganization [9]. This can cause cortical neurons that are normally sensitive to a particular auditory frequency to instead attune to other proximal frequencies [10]. Electrophysiological studies of tinnitus patients through the use of EEG and magnetoencephalography (MEG) approaches have further demonstrated that tinnitus is associated with changes in spontaneous resting-state brain activity, with one recent report indicating that tinnitus patients often exhibit enhanced baseline activity in the delta (0.5-4 Hz) and gamma (35.5-45 Hz) frequency bands and a relative decrease of alpha (8.5-12 Hz) oscillations over temporal areas as compared to healthy control individuals [11–14]. These findings were based upon the thalamocortical dysrhythmia (TCD) mode framework [15], which suggests that thalamic fibers fire spontaneously when deprived of normal external auditory input, thereby causing tinnitus [15]. This model postulates that when thalamic relay cells lose access to inner ear-derived excitatory sensory input, the hyperpolarized membrane of these cells leads to the firing of low-threshold calcium spike bursts in a slow-wave mode. This slow-wave rhythm is then established and reinforced through thalamocortical feedback loops in cortical neurons, manifesting as a form of persistent delta activity. Llinás et al. [15] further postulated that increases in gamma oscillations were responsible for tinnitus and associated perceptive disorders owing to edge effects. Inappropriate homeostatic regulation of cortical excitation and inhibition thus offers a possible explanation for the decreased alpha patterns and increased delta patterns often detected in resting-state M/EEG data from tinnitus patients [16]. TCD mode was refined by Weisz et al. to the synchronization by loss of inhibition modulation (SLIM) model, demonstrating that increased cortical gamma band activity is associated with both enhanced theta-gamma coupling as well as decreases alpha power/coherence between the MGB and A1 [17]. According to the TCD and SLIM models, the loss of feedforward input to the central auditory system (deafferentation) due to cochlear hearing loss which causes thalamocortical auditory networks to reverberate in an abnormal rhythmic pattern, resulting in the perception of sound in the absence of stimuli (i.e., tinnitus) [18].

Neurofeedback (NFB) is a noninvasive electrophysiological reading-based approach to visualizing certain brain activity parameters such that patients can alter their brain activity to negatively or positively regulate these parameters, thereby enabling patients to unlearn particular neural activity patterns related to tinnitus. NFB can be conducted using real-time EEG, MEG, or fMRI reading, with EEG-based NFB being well-established owing to its efficacy and low cost. The neural signature shown to be characteristic of tinnitus [14] helped to promote the development of novel NFB protocols aimed at treating tinnitus. Dohrmann et al. [19] designed a NFB protocol based upon these findings that sought to increase alpha activity while decreasing delta activity, although other evidence suggests that the former rather than the latter is likely to contribute to NFB-related improvements in tinnitus symptoms [20]. These reports also included small sample sizes and lacked control groups, further limiting their utility, reporting only nonspecific rising trends in the alpha/delta ratio values over the study NFB training sessions [19, 21], without sufficient analyses of posttreatment changes in brain activity. In contrast with these prior studies, we herein sought to perform a study utilizing multimodal resting-state EEG and fMRI images obtained before and after NFB training to clarify any objective changes in electrophysiological activity patterns. Nonetheless, preliminary analysis on data collected in a recent study suggested that the clinical outcome mainly correlates with the *α* band change. Therefore, we have chosen to study a NFB protocol aimed at increasing the *α* band power activity extracted from these EEG electrodes [20]. For this study, we utilized a portable EEG-mediated NFB therapy apparatus (hospital training) using alpha training protocol that had been developed by the Automation group of Chinese Academy of Sciences and our team. This training protocol was based upon self-directed regulation of the alpha frequency range, and we compared treatment outcomes in NFB patients to those in tinnitus patients not undergoing any specific treatment.

## 2. Materials and Methods

### 2.1. Participants

Participants in the present study were enrolled at the Department of Otorhinolaryngology of Chinese PLA General Hospital. Participants were eligible for inclusion if they were between the ages of 18 and 60 years old, had been diagnosed with chronic subjective tinnitus with a duration of more than 6 months, and were free of other known neurological or psychiatric disorders. Patients were also excluded from the study if they had cochlear implants, a history of drug or alcohol abuse, or were currently being prescribed tranquilizers, neuroleptics, or antiepileptics were not considered. This study was performed as a portion of an ongoing comprehensive clinical project, and patients were randomly assigned to control and NFB study groups. The control group did not receive any sham NFB training. The study was single-blinded such that only participants were not aware of their group assignments. In total, 56 patients were identified for the present study with a mean age of 46.15 ± 12.33 years (range: 18–56), including 29 males and 27 females. Of these, 38 patients were assigned to the NFB group whereas 18 were assigned to the control waitlist group. The Ethics Committee of the PLA hospital approved all study protocols detailed herein.

### 2.2. Study Procedures

Patients in the NFB group participating in this study underwent 15 visits. The first appointment was conducted 1-2 weeks prior to the start of NFB training, at which time participants were informed regarding the purpose of the study, and written informed consent was obtained in the presence of a qualified medical professional at the Department of Otorhinolaryngology. During this same visit, these individuals then underwent audiometric screening, with their pure-tone hearing thresholds at 0.125, 0.25, 0.5, 1, 2, 4, and 8 kHz, tympanograms, and other audiometric tinnitus test measurements being conducted. Average hearing thresholds were calculated by average air conduction (AC) threshold frequencies of 0.125-8 kHz. During their second visit, participants underwent resting-state EEG and fMRI analyses, and patients were instructed to complete study-related questionnaires detailed below. After these two baseline visits, participants underwent 15 total NFB training sessions over a 5-week period, with questionnaires being completed on a weekly basis. Rescheduling due to absences, illness, or holidays was conducted when possible. After the 5-week training period, EEG and fMRI measurements were repeated immediately after intervention, and patients again completed study questionnaires. Control patients also underwent audiometric screening, tinnitus tests, and behavioral measurements. During the training period, these control patients completed questionnaires on a weekly basis.

### 2.3. Behavioral Measurements

Questionnaires used to measure tinnitus patient symptoms were selected as per the Tinnitus Research Initiative (TRI) guidelines [22]. Patients were provided with an adjusted Tinnitus Sample Case History Questionnaire (TSCHQ) designed to assess their demographic information, tinnitus characteristics (origin, location, loudness, and type), prior treatments, and other tinnitus-associated complaints. A number of tinnitus questionnaires have been developed to determine the level and types of handicaps faced by tinnitus patients, including the Tinnitus Handicap Inventory (THI) and Tinnitus Handicap Questionnaire (THQ), have also been used in clinical trials to assess treatment effects. Tinnitus-related distress was assessed with the THI and THQ questionnaires, while tinnitus loudness was assessed via visual analog scale (VAS). Patients also completed the self-rating depression scale (SDS) and self-rating anxiety scale (SAS) questionnaires to exclude individuals with severe depression or anxiety who would be referred to the Psychology department. Questionnaires were completed on a weekly basis in both the control and NFB groups.

### 2.4. EEG Recording

EEG recordings were conducted twice for all 38 patients in the NFB group. Resting-state EEG data were recorded over an 8-minute period, with the first of these recordings being prior to any NFB training and the second being after the completion of all 15 training sessions. In EEG recording, the standard international system 10/20 used for electrodes' placement. The Neuroscan software (v4.5) was used to record EEG data with a 64-channel Ag/AgCl wet active electrode cap with a common vertex (Cz) reference at a 1 K Hz sampling rate. Before recordings, electrodes were checked to ensure resistance values <10 k*Ω*. Patients were directed to remain still, awake, to keep their eyes closed, and to not think of anything specific while positions in a sound- and electrically-shielded room during recording. A NeuroScan SynAmps 2 amplifier (Neurosoft, Inc., Sterling, VA) was used to amplify signals, which were filtered with a bandpass filter (BPF) at a passband of 0.1–100 Hz.

### 2.5. fMRI Data Collection

The fMRI scans were conducted on two occasions for all 38 NFB group patients using a GE 3T MR750 scanner with an 8-channel receiver array head coil. Foam padding and earplugs were used to reduce head movement and scanner-related noise. Patients were directed to remain still and awake with their eyes closed and were instructed to not think of anything specific during the scanning process. Blood oxygenation level-dependent (BOLD) signal was captured with an echoplanar imaging (EPI) sequence: repetition time (TR) = 2000 ms, echo time (TE) = 30 ms, flip angle = 90°, and 3 mm isotropic voxels. An anatomical image (FSPGR 3D sequence:

TR = 8.2 ms, TE = 3.2 ms, 1 × 1 × 1 mm resolution, 256 × 256 matrix, and flip angle = 8°) was obtained for coregistration with EPI images.

### 2.6. NFB Training

All study participants underwent 15 NFB training sessions over a 5-week period in hospital. EEG was recorded from electrodes place at frontocentral positions, for the reason of less influence from eye movement (in the training section the patients need open eyes). We choose CZ montage (in the 10-20 electrode position system). During training, electrodes were placed at central positions (Cz), with electrodes on the earlobes serving as reference electrodes and a mastoid electrode serving as a ground. EEG settings were as follows: sampling rate = 250 Hz, impedance < 20 k*Ω*. The portable EEG machine and real-time EEG signal processing was performed with a program prepared by the Automation group of the Chinese Academy of Sciences, and the resultant data were transmitted to a screen via Bluetooth. The participants were instructed to increase alpha power, with feedback being provided in the form of a rising symbol that moved from left to right across the screen. When the symbol rose higher this indicated a successful enhancement of alpha power. In addition, a smiling face icon was used to indicate when participants had reached an alpha power level above an individually adjusted threshold value. Participants were not given any specific instructions regarding how to alter EEG activity readings but were directed to alter their mental activity to achieve the desired outcome. They were instructed not to conduct any muscular activity including blinking or eye movements, as this had the potential to alter their EEG activity. Each NFB training session lasts 5 minutes. The whole sequence of recordings during a session can be summarized as RS1-Tr1-Tr2- Tr3- RS2-Tr4-Tr5.

### 2.7. Statistical Analysis

#### 2.7.1. EEG Analysis

EEGLAB (v12.0) and MATLAB (MathWorks, v R2013b) were used to process EEG data: ‘M1' and ‘M2.' Recording was referenced against the left mastoid electrode (M1) with a ground electrode positioned at the right mastoid position (M2). A notch filter was implemented at 50 Hz, and the signals were bandpass-filtered from 0.5 to 80 Hz. Gross artifacts were manually removed by visual inspection. Some artifacts originated from one or a few distinct sources or a limited volume of space were removed. In addition, some artifacts characterized by a particular temporal pattern such as exponential decay were also removed. Therefore, we use ICA algorithm to correct it. The artifact can be separated as independent components, and the other features of the original EEG signal can be effectively retained. Other artifacts, such as eye movement, muscle artifacts, and heart beats, could be also removed by independent component analysis (ICA) correction. Fast Fourier Transform (FFT) was computed for each 2 s segment, after which the data were subjected to logarithmic transformation and averaged across all segments for a given patient, with the resultant power values for each measured electrode being determined in decibels (dB) at a resolution frequency of 0.5 Hz. We use code to extract *α*-bands (8-12 Hz).

#### 2.7.2. Cortical fMRI Characteristic Calculation

Analyses of cortical thickness, surface area, volume, and curvature were examined with the DPABISurf_V1.2 software (http://rfmri.org/DPABISurf) program through an automated approach comparing T1-weighted (T1w) images before and after NFB training in all 38 patients [23]. Recon-all (FreeSurfer 6.0.) [24] was used to reconstruct brain surfaces, and the previously estimated brain mask was then refined so as to reconcile ANTs and FreeSurfer-derived segmentation of the cortical gray matter of Mindboggle [25]. Spatial normalization to the ICBM 152 Nonlinear Asymmetrical template version 2009c was conducted via a nonlinear approach using antsRegistration (ANTs 2.2.0) based upon brain-extracted T1w volume and templates.

In preprocessing, all volume slices were corrected for different signal acquisition times. Then, the time series of images for each subject were realigned. Individual structural images (T1-weighted MPRAGE) were coregistered to the mean functional image after realignment. The transformed structural images were then segmented into GM, WM and CSF. To remove the nuisance signals, the Friston 24-parameter mode was utilized to regress out head motion effects from the realigned data [26]. The signals from WM and CSF were regressed out to reduce respiratory and cardiac effects. To better control head motion effects, each bad time point with high head motion was included as a regressor. The bad time points were defined as volumes with FD (Jenkinson) > 0.2 mm as well as volumes 2 forward and 1 back from these volumes. The Jenkinson definition of FD [27] was used due to its consideration of voxelwise differences in motion in its derivation [28]. In addition, linear and quadratic trends were also included as regressors since the BOLD signal exhibits low-frequency drifts. The DPABISurf software yielded mean cortical thickness, volume, surface area, and curvature values based upon cortical atlas indices. Significant thresholds were corrected for multiple comparisons-related familywise error (FWE) via Gaussian field theory with a *p* < 0.05 clusterwise and *p* < 0.001 voxelwise confidence threshold. Functional metrics were analyzed based upon four different surface-based parameters (ALFF, fALFF, DC, and ReHo). ALFF corresponds to the mean amplitude of low-frequency FFT fluctuations (0.01–0.1 Hz) in the time course of each voxel [29]. fALFF represents a normalized ALFF determined as the total low-frequency range (0.01–0.1 Hz) divided by the overall power of the full frequency range of a given voxel [30]. DC corresponds to the number or sum of significant connection weights for each voxel. ReHo represents the homogeneity of the given voxel's time course data with that of the time course data for the 26 nearest voxels calculated based upon Kendall's coefficient of concordance (KCC) [31].

#### 2.7.3. Behavioral Measurements

SPSS v20.0 (IBM SPSS Inc., IL, USA) was used to analyze behavioral data. Demographic data were compared through Student's *t*-tests and Fisher's exact test. Paired *t*-tests were used to compare thresholds before and after NFB treatment. Data are given as means ± SD, and all statistical comparisons were two-tailed with a significance threshold of *p* < 0.05.

## 3. Results

### 3.1. Study Population

There were no significant differences between the patients in the control and NFB groups with respect to age, sex, tinnitus duration, PTA threshold, THI scores, THQ scores, or VAS scores at the time of enrollment (see Table 1).

### 3.2. Self-Reported Tinnitus Scores

**Figure 1:**
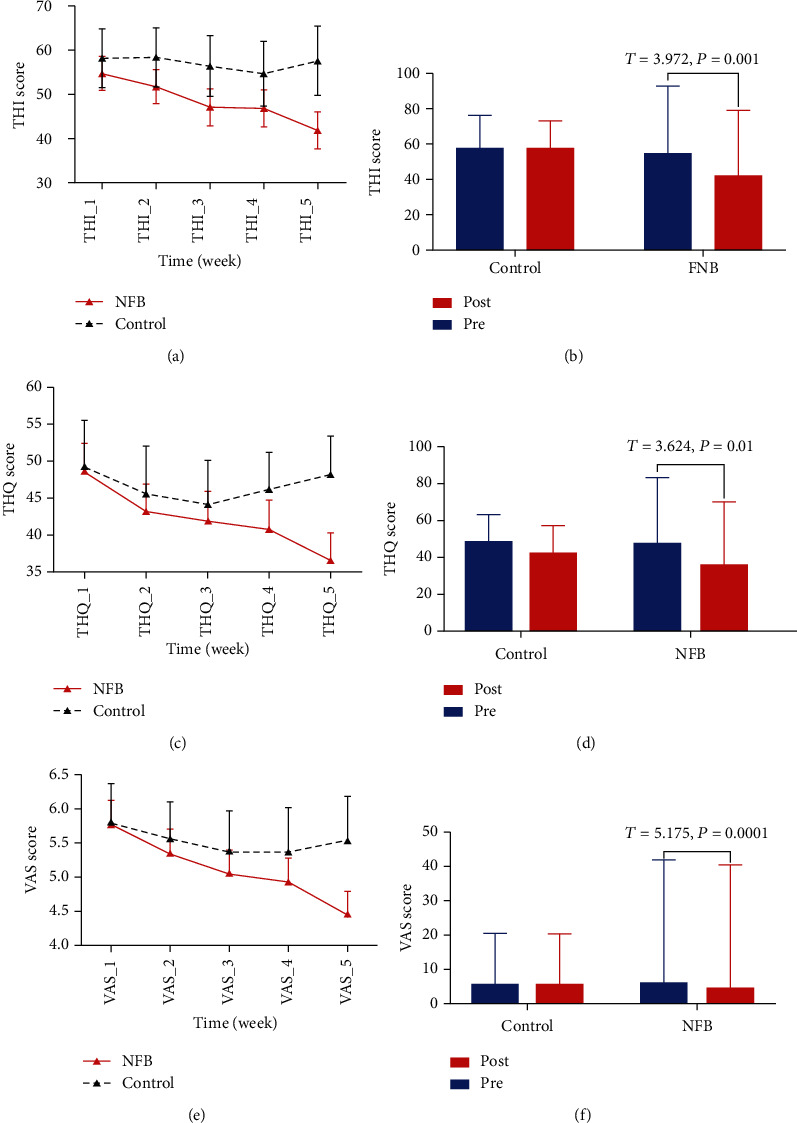
Self-reported tinnitus scores for the two groups over five weeks. The data are showed as mean ± SD. THI score of two groups for five weeks (a), comparison of pre- and posttreatment of total THI scores (b), THQ scores for the two groups over five weeks (c), comparison of pre- and posttreatment of total THQ scores (d), VAS scores for the two groups over five weeks (e), and comparison of pre- and posttreatment total VAS scores (f). NFB: neurofeedback group; control: control group.

Figure 1 and Table 2 compile the self-reported tinnitus scores from the two study groups over a 5-week period, with weekly changes in THI scores being shown in Figure 1(a), demonstrating a significant decline in THI score for participants in the NFB group without any corresponding change for controls. Only the NFB group has a statistically significant improvement in THI after treatment (Figure 1(b)). NFB group patients also exhibited a significant decline in THQ scores (Figures 1(c) and 1(d)) and VAS scores (Figures 1(e) and 1(f)) when comparing pre- and posttreatment values.

### 3.3. Electrophysiological Data Analyses

**Figure 2:**
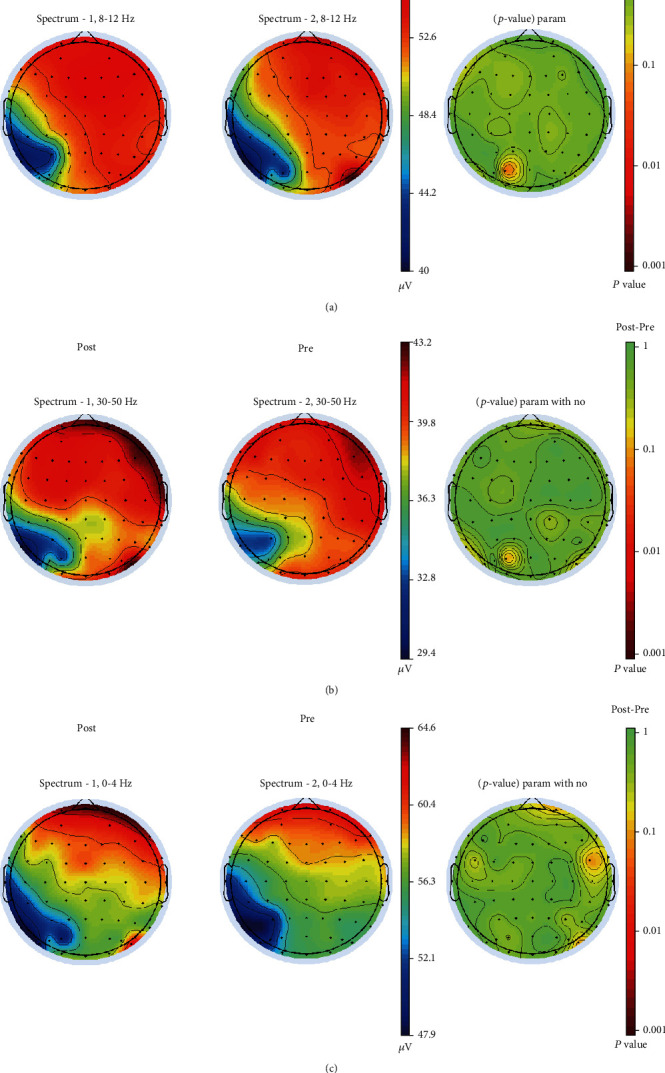
Power spectrum topographic map of the alpha, gamma, and delta bands. Power spectrum topographic map of the alpha band (a). Power spectrum topographic map of the gamma band (b) (Post: after NFB treatment, Pre: before NFB treatment, Post-Pre: Post minus Pre). Power spectrum topographic map of the delta band (c).

We compared resting-state EEG data obtained before and after the NFB intervention. With respect to frequency band specificity, the data from this study suggested specific effects in the trained frequency bands. As discussed above, the alpha band (8-12 Hz) measured over the 65 electrodes, showing that occipital alpha wave power increased and gamma, delta band power decreased (see Figure 2). Nevertheless, no significant difference remained evident after Bonferroni's correction.

### 3.4. Resting-State fMRI Data Analyses

**Figure 3:**
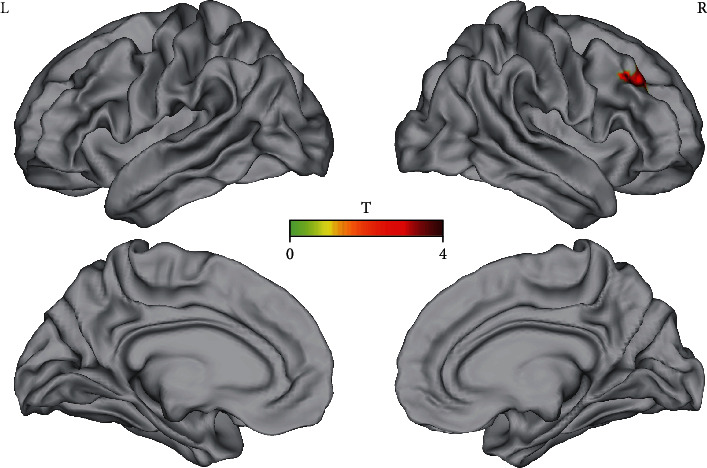
NFB-related ReHo changes. Increases in ReHo were primarily detected in the right frontal lobe. FWE correction: clusterwise threshold: *p* < 0.05 voxelwise threshold: *p* < 0.001).

ReHo values were analyzed for 38 patients before and after NFB treatment, with standard brain templates being used to display brain regions exhibiting significant differences. This analysis revealed NFB-related ReHo increases primarily within the right frontal lobe (Figure 3).

### 3.5. Cerebral Cortex fMRI Characteristics

Cortex characteristics did not differ significantly when comparing patients before and after NFB treatment.

### 3.6. Correlation Analyses

**Figure 4:**
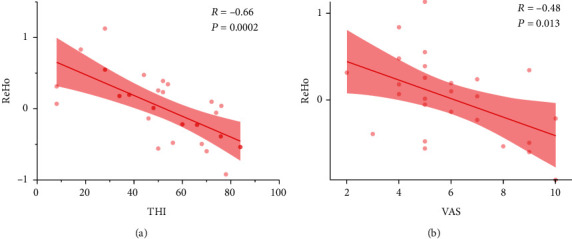
ReHo is significantly correlated with tinnitus THI and VAS scores. (a) Frontal lobe ReHo values were negatively correlated with THI scores (*R* = −0.66, *p* = 0.002). (b) Frontal lobe ReHo values were negatively correlated with VAS scores (*R* = −0.48, *p* = 0.013).

**Figure 5:**
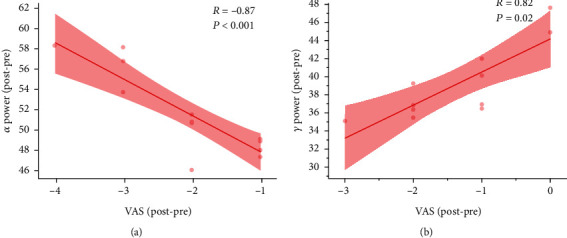
*α* and *γ* power changes were significantly correlated with tinnitus VAS scores. *α* power change were negatively correlated with VAS scores (*R* = −0.87, *p* < 0.001) (a). *γ* power change was positively correlated with VAS scores (*R* = 0.82, *p* = 0.02) (b). VAS score reduction, represented by negative values, denotes the clinical improvement of the patient condition.

In patients that underwent NFB, we found frontal lobe ReHo values to be negatively correlated with both THI scores (*R* = −0.66, *p* = 0.002) (Figure 4(a)) and VAS scores (*R* = −0.48, *p* = 0.013) (Figure 4(b)). The *α* power change was significantly correlated with tinnitus VAS scores (*R* = −0.87, *p* < 0.001) (Figure 5(a)) and *γ* power change to be positively correlated with VAS scores (*R* = 0.82, *p* = 0.02) (Figure 5(b)). EEG changes did not significantly correlated with the ReHo changes in the right frontal lobe.

## 4. Discussion

In the present study, we employed a clinical NFB protocol aimed at alpha training which we found to significantly benefit tinnitus-related distress (THI and THQ scores) and tinnitus loudness (VAS scores) in treated patients. Our data are consistent with those from two prior studies of NFB protocols (alpha/delta training protocols) that also reported significant improvements in THQ and THI scores following the training period [21]. Given the consistency of these findings, it is likely that augmenting alpha activity can recapitulate the benefits of protocols that increase alpha activity and decrease delta activity, underscoring the notion that NFB-related changes in symptoms of tinnitus are primarily linked to increase alpha activity and not to reduce delta power [20, 32].

As traditional NFB approaches use relatively few surface electrodes, they have often been criticized for their poor spatial specificity. However, one prior study randomly assigned patients to undergo either tomographic (TONF) or traditional electrode-based neurofeedback (NTNF) and observed comparable significant improvements in tinnitus symptoms and loudness in both groups, suggesting that both approaches are efficacious [33]. Given that surface electrodes are more convenient to employ in a clinical setting, we therefore utilized electrode-based NFB in this study. Dohrmann et al. [19] and Crocetti et al. [21] previously reported NFB protocols wherein treatment was conducted 2-3 times per week for 30 and 20 minutes, respectively, revealing 30-minute sessions to be more effective than 20-minute sessions. Both training frequency and session length were thus both speculated to be critical to achieving sustained benefits for tinnitus patients. We therefore conducted 30-minute NFB treatment sessions three times per week, revealing significant improvements in tinnitus loudness (VAS scores).

Analyzing electrophysiological data offers an effective means of determining whether NFB treatment alters neurological activity patterns in treated tinnitus patients. Neither Dohrmann et al. [19] nor Crocetti et al. [21] conducted any multimodal neuroimaging (resting-state EEG or fMRI) before and after training, instead focusing on data during the training phase before and after individual training sessions. In so doing, they primarily detected nonspecific upward trends in alpha/delta ratios during training. In contrast, we conducted multimodal analyses of patients in the NFB group before and after the intervention period in order to gain more insight into objective treatment-related changes in electrophysiological activity patterns. Resting-state EEG data comparisons revealed that trained alpha-band activity was significantly increased following the 15 treatment sessions, consistent with successful frequency pattern establishment. A significant increase was found for EEG pre- and post-NF treatment in the alpha band at occipital in which alpha band was more obvious that brain area [34]. However, these differences were not significant following Bonferroni correction, likely due to the small sample size in this study. Even so, these results suggest substantial stable increases in the alpha band across the study period, providing a valuable indication that trained frequency patterns were established. As EEG offers low spatial resolution, it was not sufficient to establish the specific changes associated with NFB treatment. We therefore conducted high-resolution resting-state fMRI analyses to more specifically localize these changes using an innovative surface-based morphometry (SBM) approach that is superior to traditional value-based management (VBM) approaches with respect to overall accuracy [35]. Consistent with prior studies [36–38], we found NFB therapy to improve tinnitus-associated distress (THI scores) and loudness (VAS scores) through mechanisms correlated with increased frontal lobe activity (Figures 3 and 4). The frontal lobe is important for managing emotions, and following NFB treatment patients may be better able to utilize the frontal lobe to manage their emotional responses to tinnitus, thereby reducing perceived tinnitus distress and loudness. Frontal lobe activity was significantly negatively correlated with patient THI and VAS scores, suggesting that higher levels of such activity are linked to better patient outcomes. Given that frontal lobe activity was increased following treatment, this further supports the efficacy of this NFB-based approach. No treatment-related changes in cortical characteristics were observed, likely owing to the relatively short treatment duration. In conclusion, we found that alpha NFB training was sufficient to increase frontal lobe excitability and to thereby improve tinnitus patient symptoms.

There are certain limitations to the present study that should be taken into consideration. The waiting list group as the control group may affect the results due to psychological effects. The exact control group is needed for future study, same as Vanneste's study [32]. For one, our control (*n* = 18) and NFB (*n* = 38) groups were not equally sized, and this has the potential to impact statistical power and homogeneity of variance assumptions. However, as our power calculations were based upon the smallest sample size, such unequal grouping is likely to have improved our ability to reliably differentiate between these two groups. Furthermore, our study was single-blinded such that only participants were not aware of their group assignments. This has the potential to have introduced unintentional bias into our results as a consequence of unconscious researcher demands on these two groups, which may have influenced participant brain activity and that control group had no sham feedback or active nonneurofeedback. As such, future research will be needed to confirm the reliability of these results.

## 5. Conclusions

The results of this analysis indicate that alpha NFB training can be effectively used to reduce tinnitus-related distress (THI and THQ scores) and sound perception (VAS scores) in patients. Functional imaging changes further reveal that NFB treatment can increase alpha wave power in the occipital lobe and ReHo values in the frontal lobe in a manner correlated with improvements in tinnitus patient symptoms.

## Figures and Tables

**Table 1:** Patient characteristics.

Group	NFB (*n* = 38)	Control (*n* = 18)	*p* value
Age (years)	47.91 ± 18.36	45.50 ± 10.72	0.350
Gender (male : female)	19 : 19	10 : 8	0.877
Tinnitus duration (months)	6.94 ± 3.85	3.4 ± 3.06	0.631
PTA threshold (dBHL)	27.26 ± 13.8	29.53 ± 12.9	0.396
THI total score (first time)	54.78 ± 15.7	58.28 ± 17.3	0.271
THQ (first time)	48.72 ± 11.9	49.36 ± 10.8	0.392
VAS (first time)	5.71 ± 2.20	5.76 ± 1.92	0.523

Abbreviation: NFB: neurofeedback; dBHL: decibel hearing level. Data are means ± SD.

**Table 2:** Self-reported tinnitus scores for the two groups after NFB treatment.

	NFB_post	NFB_pre	Control_post	Control_pre	*p* value
THI	41.90 ± 31.26	54.79 ± 30.23	57.62 ± 15.38	58.28 ± 18.14	0.001
THQ	36.57 ± 15.13	47.38 ± 18.16	42.86 ± 14.12	48.97 ± 14.37	0.01
VAS	4.46 ± 4.01	5.80 ± 4.93	5.53 ± 4.36	5.9 ± 5.10	0.0001

Abbreviation: NFB: neurofeedback. Data are means ± SD.

## Data Availability

Data is available on request.
